# Exploring the immunopathology of type 2 inflammatory airway diseases

**DOI:** 10.3389/fimmu.2024.1285598

**Published:** 2024-04-12

**Authors:** Shaimaa AlBloushi, Mona Al-Ahmad

**Affiliations:** ^1^ Al-Rashed Allergy Center, Ministry of Health, Kuwait City, Kuwait; ^2^ Microbiology Department, College of Medicine, Kuwait University, Kuwait City, Kuwait

**Keywords:** CRS, type 2 inflammation, CRSwNP, AERD, AFRS, EGPA, ABPA, eosinophilic asthma

## Abstract

Significant advancements have been achieved in understanding the roles of different immune cells, as well as cytokines and chemokines, in the pathogenesis of eosinophilic airway conditions. This review examines the pathogenesis of Chronic Rhinosinusitis with Nasal Polyps (CRSwNP), marked by complex immune dysregulation, with major contributions from type 2 inflammation and dysfunctional airway epithelium. The presence of eosinophils and the role of T-cell subsets, particularly an imbalance between Treg and Th17 cells, are crucial to the disease’s pathogenesis. The review also investigates the pathogenesis of eosinophilic asthma, a unique asthma subtype. It is characterized by inflammation and high eosinophil levels, with eosinophils playing a pivotal role in triggering type 2 inflammation. The immune response involves Th2 cells, eosinophils, and IgE, among others, all activated by genetic and environmental factors. The intricate interplay among these elements, chemokines, and innate lymphoid cells results in airway inflammation and hyper-responsiveness, contributing to the pathogenesis of eosinophilic asthma. Another scope of this review is the pathogenesis of Eosinophilic Granulomatosis with Polyangiitis (EGPA); a complex inflammatory disease that commonly affects the respiratory tract and small to medium-sized blood vessels. It is characterized by elevated eosinophil levels in blood and tissues. The pathogenesis involves the activation of adaptive immune responses by antigens leading to T and B cell activation and eosinophil stimulation, which causes tissue and vessel damage. On the other hand, Allergic Bronchopulmonary Aspergillosis (ABPA) is a hypersensitive response that occurs when the airways become colonized by aspergillus fungus, with the pathogenesis involving activation of Th2 immune responses, production of IgE antibodies, and eosinophilic action leading to bronchial inflammation and subsequent lung damage. This analysis scrutinizes how an imbalanced immune system contributes to these eosinophilic diseases. The understanding derived from this assessment can steer researchers toward designing new potential therapeutic targets for efficient control of these disorders.

## 1 Introduction

Chronic rhinosinusitis (CRS) exerts a substantial influence on public health and socio-economics due to its diverse nature and complex underlying mechanisms (1). The prevalence of CRS in the general population ranges from 5% to 15%, indicating its widespread occurrence (2). CRS is defined by the presence of continuous inflammation in the paranasal sinuses that endures for a minimum duration of 12 weeks (3). It is primarily classified into two categories: Chronic Rhinosinusitis with Nasal Polyps (CRSwNP) and Chronic Rhinosinusitis without Nasal Polyps (CRSsNP), with additional sub-phenotypes such as cystic fibrosis and aspirin-exacerbated respiratory disease (4).

Chronic Rhinosinusitis with Nasal Polyps (CRSwNP), a persistent inflammatory ailment impacting the nasal mucosa and paranasal sinuses, substantially diminishes the quality of life and results in morbidity (1, 5). Moreover, CRS particularly CRSwNP is highly prevalent in individuals with asthma, and its presence is linked to poorer asthma outcomes (5–7). Additionally, a strong correlation has been identified between asthma and rhinosinusitis, and the development of nasal polyps (7).

Asthma displays a wide spectrum of clinical symptoms among individuals, accompanied by diverse inflammatory mechanisms that play a role in the onset, continuation, and severity of the condition. This heterogeneity allows to divide asthma into asthma type 2 and non-type 2 respectively (8, 9). Asthma type 2 has further subtypes including eosinophilic asthma that is characterized by elevated eosinophil counts in both tissues and blood (10, 11) Approximately half of individuals with asthma experience eosinophilic inflammation. Research has demonstrated a connection between eosinophilia and heightened disease severity, more frequent exacerbations, greater symptom burden, and impaired lung function (12, 13).

The underlying immune processes responsible for both eosinophilic asthma and CRSwNP have been well-established and, are characterized by type 2 inflammation (8, 14). Within the airway, type 2 immune responses result in the collection of eosinophils, mast cells, basophils, Th2 cells, type 2 innate lymphoid cells (ILC2s), and B cells that produce IgE. These immune cells, along with type 2 mediators and cytokines (such as IL-4, IL-5, and IL-13), have been identified as contributing factors in the development of type 2 associated pathologies including CRSwNP and eosinophilic asthma (8, 15, 16).

The understanding that both diseases are influenced by immune system dysregulation is now widely acknowledged (17). Hence, acquiring a thorough comprehension of the immune mechanisms at play offers the potential for pinpointing therapeutic targets. Consequently, this review aims to explore the involvement of the dysregulated immune system in the progression of CRSwNP, eosinophilic asthma, and similar conditions such as eosinophilic granulomatosis with polyangiitis (EGPA) and allergic bronchopulmonary aspergillosis (ABPA). The review intends to provide valuable insights to researchers, facilitating the identification and development of potential drug targets for the management of these conditions.

## 2 Immunopathology of chronic rhinosinusitis with nasal polyps according to endotypes

The available data on immune system dysregulation in CRSwNP is variable. Although the inflammatory environment in CRSwNP has been extensively studied, the specific triggers and signals that initiate this immune response remain poorly defined (18, 19). Therefore, the exact mechanisms underlying immune dysregulation in CRSwNP are not fully understood, but several factors are believed to contribute to this condition (20–22). Within the broad category of Chronic Rhinosinusitis (CRS) characterized by a diffuse type 2 inflammatory response, there exist two distinct phenotypes: Central Compartment Atopic Disease (CCAD) and Eosinophilic Chronic Rhinosinusitis (eCRS). According to the EPOS 2020 guidelines, both CCAD and eCRS are identified by a type 2 inflammatory pattern, with a histological marker of increased eosinophils in tissue, quantified as more than 10 eosinophils per high power field (23). The differentiation of these two eosinophilic subtypes is primarily based on clinical assessment, endoscopic findings, and radiological evidence rather than on histological data alone. Other classifications within CRS with Nasal Polyps (CRSwNP) encompass Allergic Fungal Rhinosinusitis (AFRS) and Aspirin-Exacerbated Respiratory Disease (AERD).

Central Compartment Atopic Disease (CCAD) is recognized as a subtype of Chronic Rhinosinusitis (CRS) observed primarily in patients with high allergic sensitivity. The initial precursor of CCAD is the presence of middle turbinate polypoid edema and the disease progresses with discrete polyps. Additional characteristics of CCAD include obstructive edema, formation of polyps, and increased soft tissue density within the central area of the sinonasal region, typically without affecting the outer walls, as determined through endoscopic and radiological evaluations (24). The younger population is more prone to this phenotype and airborne allergens are closely linked with this condition necessitating targeted immunotherapy for effective management (24, 25). Moreover, individuals with CCAD have elevated levels of interleukin-5 and interleukin-13 (26). Edwards and colleagues (27) discovered that among their 15 study participants, 14 exhibited sensitivity to at least one allergen detected locally in the central compartment and through systemic skin or serum tests. Four participants reacted to allergens on central compartment-specific IgE (sIgE) tests that did not provoke a response in skin and serum sIgE tests, revealing a range of statistically significant correlation strengths from weak to strong.

Eosinophilic Chronic Rhinosinusitis (eCRS) is recognized as a distinct variant of Chronic Rhinosinusitis (CRS) that manifests as nasal polyps, predominantly occurring in the middle-aged population with a strong association with the onset of asthma in adulthood, often among individuals without a significant history of sinus disease or allergies. Endoscopic examination typically reveals widespread polyp formation and dense, eosinophil-rich mucin that has a sticky consistency reminiscent of “chewing gum” (28). Furthermore, the mucin exhibits distinctive histopathological characteristics, including the presence of eosinophils, clusters of eosinophils, and Charcot-Leyden crystals - all valuable indicators for diagnosis and further examination (28). According to findings from the Japanese Epidemiological Survey of Refractory Eosinophilic Chronic Rhinosinusitis (JESREC), there is a significant correlation between the presence of IgE-positive mast cells within the nasal mucosa and the clinical severity as well as histopathological factors in eCRS (29). Compared to Central Compartment Atopic Disease (CCAD), individuals with eCRS exhibit considerably higher levels of eosinophils and eotaxin-3 in their tissues, underscoring a significant immunological distinction between the two subtypes (29). Elevated systemic eosinophil counts are characteristic of eosinophilic Chronic Rhinosinusitis (eCRS). An increased eosinophil count in the bloodstream is indicative, with a positive likelihood ratio (LR) of 3.28, of predicting substantial eosinophil presence within tissues. However, the occurrence of eosinophils within tissues does not show a significant correlation with the levels of specific IgE to allergens found in serum (30).

When distinguishing between Central Compartment Atopic Disease (CCAD) and Eosinophilic Chronic Rhinosinusitis (eCRS), it is important to evaluate a range of factors, including the age of onset (early adulthood for CCAD versus middle age for eCRS), the location of polyp growth as determined by endoscopy (nasal turbinates for CCAD versus sinuses for eCRS), the spread of radiological findings (central for CCAD versus diffuse for eCRS), and the degree of eosinophilic infiltration in tissues. Other considerations include when related respiratory conditions begin (childhood for CCAD versus adulthood for eCRS) and the timing of the loss of smell (typically occurring later for CCAD compared to earlier for eCRS) (29). The presence of concurrent allergic conditions is also an important distinguishing factor. Kong et al. identified that a significantly larger segment of the CCAD group, 57.14%, experienced allergic rhinitis (AR) in contrast to 21.88% within the eCRS group. Sensitivity to inhaled allergens was universally present in the CCAD cohort, often to indoor allergens such as dust mites, cockroaches, and pet allergens. Additionally, the peripheral eosinophil percentage in CCAD patients tends to be moderately lower than that in individuals with eCRS (31).

Aspirin-Exacerbated Respiratory Disease (AERD) is another phenotype of CRSwNP. Genetic variations and epigenetic malfunctions might be implicated in causing AERD. These changes can influence the dynamics of enzymes and the responsiveness of receptors, leading to an escalation in leukotriene (LT) synthase activity and an abundance of cysteinyl leukotrienes (cysLTs). An increase in the sensitivity of LT receptors, along with a surge in cysLT receptor 1 expression, is observed. Concurrently, the production of prostaglandin E2 sees a decline, alongside a decrease in the activity of cyclooxygenase-2 (COX-2) and E-prostanoid receptor subtype-2, potentially aggravating the eicosanoid imbalance (32). The intricate nature of inflammatory mediators in AERD is highlighted by the disruption of the PG2-dependent regulation of LT production in peripheral granulocytes. This underscores the complexity that researchers and medical professionals face when studying this condition. Patients with AERD exhibit remarkable differences in their granulocytes compared to those with ASA-tolerant asthma or control subjects. Notably, these granulocytes generate higher levels of LTB4 and cysLTs, while displaying resistance to the usual PGE2-mediated suppression of LT generation. This unique characteristic highlights the distinct nature of AERD and further underscores the importance of understanding its underlying mechanisms. The reason behind this phenomenon can be attributed to a malfunctioning protein kinase A in AERD. This malfunction can disrupt the normal regulation of 5-lipoxygenase activity by PGE2, causing an imbalance in control mechanisms (32). The understanding of AERD has greatly benefited from the exploration of pathophysiological and immunological factors, which have now become crucial in our knowledge. As a result, the development of *in vitro* tests has emerged as a significant advancement in this field. These tests have the potential to evolve into a viable alternative for identifying a significant subgroup of CRSwNP patients, replacing traditional provocation testing (32).

AERD’s inflammatory complexity is underscored by altered prostaglandin E2 control over LT production in peripheral granulocytes. When compared to granulocytes from individuals with aspirin-tolerant asthma or those who are healthy, those from AERD patients show higher levels of leukotriene B4 and cysLTs and a reduced suppression of LT production by PGE2. This could be attributed to a compromised protein kinase A function in AERD, affecting the regulation of 5-lipoxygenase by PGE2 (33).

Beyond the established type 2 inflammation signature in AERD, the significance of innate immune reactions is becoming more recognized. Type 2 innate lymphoid cells and heightened levels of IL-33 and thymic stromal lymphopoietin (TSLP) are noted for their role in amplifying the activity of lymphoid and myeloid cells, particularly mast cells. Both IL-33 and TSLP are abundantly present in nasal polyp tissues and are influential in the inflammatory pathways as demonstrated in non-human studies (34). Furthermore, it was also found that the expression of IL-25, IL-33, TSLP, and RANK-L is comparable between patients with CRSwNP alone and AERD (34). This finding is significant in understanding the similarities between these two conditions. **Table 1** summarizes the similarities and differences between CRSwNP endotypes.

**Table 1:** Compares the four endotypes of Chronic Rhinosinusitis with Nasal Polyps (CRSwNP) including Central Compartment Atopic Disease (CCAD), Eosinophilic Chronic Rhinosinusitis (eCRS), Allergic Fungal Rhinosinusitis (AFRS), and Aspirin-Exacerbated Respiratory Disease (AERD), detailing the predominant type of inflammation, key cells involved, cytokines, and the role of eosinophils in each endotype.

Endotype	Predominant Type of Inflammation	Key Cells Involved	Role of Eosinophils	Cytokines and Chemokines	IgE Level	Associated Conditions
CCAD	Type 2	Eosinophils, mast cells	Increased in tissue, related to allergy	IL-5, IL-13	Elevated in most to all patients, can have local allergen sensitivity only	Allergic rhinitis, allergy to indoor allergens
eCRS	Type 2	Mainly Eosinophils, mast cells, IgE-positive cells	Increased in tissue, high systemic count	IL-5, eotaxin-3, IL-13, IL-33, TSLP	Elevated with specific IgE to allergens in serum	Asthma onset in adulthood
AFRS	Type 2	Eosinophils, mast cells	Main contributors to inflammation	IL-4, IL-5, IL- 13	High levels of local and serum IgE to aspergillus fumigatus	Hypersensitivity to fungal antigens
AERD	Type 2	Granulocytes (eosinophils, neutrophils)	Involved in propagation of inflammation	IL-33, TSLP, (increased LT production)	Comparable to CRSwNP alone	Aspirin sensitivity, increased leukotriene activity

Allergic fungal rhinosinusitis (AFRS) is a form of chronic rhinosinusitis predominantly driven by a Th2 immune response. It’s diagnosed based on specific criteria established by Bent and Kuhn. The distinguishing features of Allergic Fungal Rhinosinusitis (AFRS) include the existence of fungal growth, the presence of IgE antibodies, and systemic hypersensitivity to fungal antigens. Early research on the causes of AFRS has confirmed this theory by detecting specific IgE and IgG antibodies against fungi in patients with AFRS. The scrutiny surrounding this issue escalated as subsequent studies revealed the existence of beneficial fungal cultures in patients with non-allergic fungal rhinosinusitis (AFRS), as well as in those who were healthy. This has raised important questions and calls for further investigation into the implications and potential benefits of these findings. Diagnosis hinges on detecting systemic IgE antibodies against fungal antigens, distinguishing AFRS from eosinophilic fungal rhinosinusitis. Fulfilment of major Bent and Kuhn criteria includes nasal polyps, evidence of fungi in stains, eosinophilic mucin without fungal tissue invasion, characteristic CT imaging showing varied soft tissue densities, and type I hypersensitivity to fungi. Additional minor criteria encompass the presence of eosinophil-rich allergic mucin identifiable visually or via histopathology, positive fungal staining, and the absence of immunodeficiency or diabetes. AFRS constitutes approximately 6–9% of cases of fungal rhinosinusitis (35). The research mentioned above suggests that the pathophysiology of AFRS is quite similar to other subtypes of CRS, rather than being significantly different. However, it is worth noting that this conclusion may be due to a lack of studies systematically examining the molecular profiles of AFRS in comparison to other subtypes. Orlandi et al. conducted a comprehensive microarray gene expression analysis to uncover the differences in gene expression between AFRS and eCRS (36). Through their research, they identified four genes that were specifically overexpressed in eCRS but not in AFRS, as well as 34 genes that exhibited unique overexpression in AFRS. This study sheds light on the distinct genetic patterns associated with these two conditions. The study’s findings may be influenced by its small sample size, as it only involved a total of 7 patients. Additionally, the rationale for evaluating the target genes under study was not clearly explained, which could affect the overall reliability of the results.

Tyler and team discovered distinct variations in gene expression specific to Allergic Fungal Rhinosinusitis (AFRS), significantly related to Th2 inflammation, enhanced co-stimulatory signaling, and T-cell receptor dynamics (37). This is supported by the association of AFRS with well-known costimulatory and T-cell receptor signaling pathways, as revealed through pathway analysis. These findings offer valuable insights into the biological foundation of the “allergic type” inflammatory signature. They shed light on the excessive production of mucus, hypersensitivity to fungi, tissue eosinophilia, and robust local and serum IgE production. Without a doubt, the expression of IL-4, IL-5, and IL-13 can be attributed to various sources such as T cells, mast cells, and innate lymphoid cells when they respond to a wide array of signals originating from both the innate and adaptive immune systems. The findings suggest that the adaptive immune system plays a crucial role in distinguishing AFRS and is a powerful contributor to type 2 inflammation (37). Additionally, they noted that patients with AFRS have heightened levels of IL-33 receptors, which correlate with an uptick in gene expression for eosinophils and mast cells (38).

Den et al., observed that cells from AFRS patients showed a depletion in proteins that maintain tight junction integrity, alongside a rise in proteins like claudin-2 that contribute to junction permeability (39). The observed changes in claudin-2 expression, accompanied by decreases in JAM-A and occludin, strongly suggest that a significant mechanism involving transcriptional-level protein alteration is responsible for the restructuring of tight junctions in AFRS (39). This finding provides a comprehensive explanation for the observed remodeling process.

In another study by Tyler et al., it was found that while antifungal histatin peptides are typically present in the major salivary glands, they are also found in the sinonasal mucosa of individuals with Chronic Rhinosinusitis with Nasal Polyps (CRSwNP), but these peptides are absent in AFRS affected tissues. This study also indicated that histatin levels inversely correlate with Type 2 inflammatory mediators such as IL-13RA1, IL-4R, and periostin (37). Ebert et al. utilized a microarray to demonstrate that AFRS tissues have a higher expression of a Th2-promoting receptor compared to those from healthy subjects, suggesting a potential role in the disease’s inflammatory response (40). A shortfall in the Th17 immune response in AFRS has been noted, which is crucial in combating bacterial and fungal infections that target the epithelial barrier. **Table 1**. Compares the key features of the four endotypes of Chronic Rhinosinusitis with Nasal Polyps (CRSwNP).

### 2.1 Epithelial barrier dysfunction

The airway epithelium plays a vital role in innate and adaptive immunity, serving as a defense mechanism against inhaled particles (41). When the respiratory epithelium is compromised, it can trigger immune and inflammatory responses, resulting in chronic inflammation observed in conditions like chronic rhinosinusitis (42).

In the case of CRSwNP, there is evidence of dysfunction in the epithelial barrier, affecting various aspects such as mucociliary clearance, cytokine secretion, and modulation of immune cells (43). Additionally, nasal polyps from CRSwNP patients show less expression of certain adhesion molecules compared to normal mucosal tissue (44). The underlying mechanisms responsible for epithelial cell dysfunction in nasal polyps are not fully understood (20).

Based on current literature, the dysfunction involves disrupted inflammatory pathways and alterations in airway lining cells (43, 45). The study by Wang et al. (2015) revealed that in the context of epithelial barrier alteration, there is a notable occurrence of hyperplasia in basal cells and goblet cells. Additionally, it was observed that ciliated columnar cells exhibited impaired cilia architecture and elevated protein expression levels of markers associated with ciliogenesis (46). These issues have been linked to various cytokines. For instance, IFN-γ and IL-13 were found to significantly affect the development and motility of ciliated cells, as well as the presence of goblet cells and the expression of MUC5AC mucin in CRSwNP patients. Furthermore, IL-17 exhibited a significant association with increased MUC5B mucin expression (47). In addition to these factors, communication with mast cells also plays a role, contributing to persistent type 2 inflammation (43, 45).

### 2.2 Type 2 inflammation

It is now widely acknowledged that CRSwNP is linked to type 2 inflammation. This form of inflammation signifies an intricate immune reaction (29). When the innate immune system is stimulated, it leads to the induction of type 2 innate lymphoid cells (ILC2) (8, 34). In nasal polyps, ILC2s display robust activation and actively generate type 2 cytokines within the tissue (34). On the other hand, the adaptive immune system becomes triggered upon allergen exposure, resulting in the stimulation of type 2 T-helper (Th2) cells. Both ILC2 and Th2 cells release type 2 cytokines, such as interleukin-4 (IL-4), interleukin-5 (IL-5), and interleukin-13 (IL-13), which fulfil various functions within the inflammatory process. These interleukins play a role in attracting eosinophils to tissues, resulting in the distinctive clinical symptoms observed in chronic inflammatory airway diseases (8). Consequently, the presence of pronounced tissue eosinophilia is notably linked to type 2 inflammation in CRSwNP (19, 48). Furthermore, the shared mechanism of type 2 inflammation is one of the contributing factors to the coexistence of CRS and specifically CRSwNP with asthma (49, 50). Due to this, a remarkably high prevalence rate of 66% to 69% was documented among individuals with comorbidities of type 2 asthma and CRSwNP (51).

While the involvement of ILC2s, Th2 cells, and eosinophils in type 2 inflammation associated with CRSwNP is evident, ongoing research is exploring the contribution of epithelial cells, macrophages, and neutrophils to the development of CRSwNP (52).

### 2.3 Role of eosinophils in CRSwNP

CRSwNP also encompasses a disruption of the innate immune system (36, 37), resulting in an exaggerated and prolonged inflammatory response (53). In this instance, eosinophils take on the central role among the inflammatory cells contributing to CRSwNP (54, 55). Due to the involvement of eosinophils in CRSwNP a new term “eosinophilic chronic rhinosinusitis” was first coined in 1990 following a survey conducted on patients diagnosed with CRS accompanied by nasal polyps. The survey results revealed that these patients primarily experienced a change in their sense of smell, along with the presence of thick nasal discharge and nasal blockage. Noteworthy, the nasal polyp tissues obtained from these patients exhibited a remarkable infiltration of eosinophil-dominant inflammatory cells, in contrast to the previously more common neutrophil-dominant infiltration observed (56).

Various mechanisms have been identified by researchers to explain the occurrence of eosinophilic infiltration in CRSwNP (57, 58). It is evident that in CRSwNP, there is an accumulation of Charcot-Leyden Crystals (CLCs) within the tissues, as indicated by previous research (42). CLCs are exclusively composed of galectin-10, which is a highly abundant protein located in the eosinophil cytoplasm (43). It has been consistently observed that CLC deposition tends to occur alongside an excessive infiltration of eosinophils. Notably, in the context of CRSwNP, the presence of galectin-10 protein and CLCs is more commonly associated with type 2 inflammation, suggesting a potential marker for heightened eosinophil activation (42). Th2 cells are also responsible for secreting IL-5, a cytokine that serves to activate and attract eosinophils too (59).

Asides from eosinophils, emerging evidence also suggests that ILC2s have a central role in the commencement, persistence, and propagation of conditions characterized by type 2 airway inflammation (2). The outcomes of a recent study revealed a connection between CRSwNP with eosinophilic inflammation and an increase in both the quantities of ILC2s and the production of IL-5 by ILC2s, particularly in individuals concurrently diagnosed with asthma. Conversely, a significant correlation has been observed between the prevalence of ILC2s and IL-5-producing ILC2s and the concentrations of IL-5, CCL24, and IgE identified within nasal polyp tissues. These results imply that ILC2s may potentially play a role in the onset of eosinophilic inflammation in CRSwNPs. Furthermore, the sinus mucosa harbors ILC precursors that possess the capacity to differentiate into either interferon-gamma (IFN-γ)-producing cells or IL-5-producing cells, contingent upon the local cytokine they encounter (60). These cytokines include IL-4, IL-5, IL-13, and IL-9 (2).

### 2.4 Altered T-cell response

T-cells, including Th1, Th2, Th17, and regulatory T-cells (Tregs), are essential for immune regulation (61). Treg cells are a specific subset of T cells with immunoregulatory functions. The malfunction and inadequate differentiation of Treg cells have a significant impact on the initiation and progression of CRSwNP (62). The complexity of this mechanism has been amplified by the discovery of additional roles of subsets of T helper (Th) cells, such as Th22, Th9, and follicular Th cells, as well as regulatory B cells in CRSwNP (63).

To summarize, CRSwNP is marked by a Th2 immune response, alongside an imbalance between Treg and Th17 cells in the polyp tissue. There are reduced Treg levels and increased Th17 levels (62). The colonization of certain pathogenic bacteria and fungi has been linked to nasal polyp development in CRSwNP (64, 65). This colonization is interrelated with T helper 2 (Th2) inflammation. Furthermore, Individuals experiencing nasal polyposis exhibit a diminished presence of CD4+ T cells and an augmented prevalence of CD8+ T cells. Notably, both nasal CD8+ T cells and CD4+ T cells predominantly demonstrate an effector memory phenotype. More specifically, there was an observed increase in activated Treg cells within the CD4+ T cell population in nasal polyps compared to Peripheral Blood Mononuclear Cells (52). The recruitment of CD4+ T cells into the nasal mucosa is under the influence of regulatory T (Treg) cells, which are thought to play a pivotal role in the formation of nasal polyps by modulating the equilibrium between Th1 and Th2 immunity (66).

### 2.5 Genetics in CRS w NP

Polymorphisms in the gene coding for the bitter taste receptor T2R38 are linked to a heightened susceptibility to Chronic Rhinosinusitis (CRS). Furthermore, in his study, Cohen showed that individuals with nonfunctional T2R38 alleles (AVI) have decreased nitric oxide and ciliary responses in their upper airway epithelial cells when exposed to gram-negative quorum sensing molecules. This makes them more susceptible to infections from gram-negative bacteria like Pseudomonas aeruginosa and increases the likelihood of developing chronic rhinosinusitis requiring surgery (67).

## 3 Immunopathology of eosinophilic asthma

Eosinophilic asthma is a distinct form of asthma characterized by inflammation of the basement membrane in the airway mucosa and elevated levels of eosinophils in sputum and blood (22, 59). Patients with asthma and blood eosinophil counts ≥150 cells/mL have a higher risk of hospitalization, at least 1.3 times greater than those with counts below this threshold (68).

a Few conditions present as asthma with eosinophilia, such as allergic bronchopulmonary aspergillosis, aspirin-exacerbated respiratory disease, allergic and non-allergic types etc. Despite these variations, the underlying immunopathology generally remains consistent (10, 69, 70). In general, eosinophilic asthma arises from interactions among genetic factors, environmental factors, and the immune system, involving type 2 inflammation (71).

Dendritic cells residing in epithelium have a crucial function in capturing allergens, processing them, and presenting antigens to naive T helper cells (Th0). Exposure to antigens triggers Th0 cells, causing them to undergo differentiation into Th2 cells and subsequently release cytokines such as IL-4 and IL-13 (58). This activation of Th2 cells stimulates class switching in B cells, resulting in the synthesis of IgE immunoglobulin. Moreover, Th2 cells secrete IL-5, which serves to activate and attract eosinophils (56). In response to IgE, mast cells release a range of mediators including leukotrienes, prostaglandins, and cytokines, contributing to immediate and newly developed allergic reactions (59). Similarly, Type 2 innate lymphoid cells (ILCs2) generate IL-13 and IL-5, which also participate in the recruitment and proliferation of eosinophils, particularly in non-allergic eosinophilic asthma (eBA) (70). Understanding the immunopathology of eosinophilic asthma is essential for developing specific therapies to manage this condition effectively (72). The upcoming sections will provide insights into the immunopathological mechanisms involved in eosinophilic asthma.

### 3.1 Pollutants as a risk factor for asthma

Atmospheric contaminants are believed to be a factor in the emergence of eosinophilic types of airway disease beginning in adulthood. The longitudinal research by Pepels and colleagues established an association between the presence of nitrogen dioxide (NO2), ozone (O3), and black carbon in the environment and the development of Airway Disease. This link did not extend to fine particulate matter (PM2.5). It was observed that the association was more pronounced for eosinophilic Airway Disease than for its non-eosinophilic counterpart (73).

### 3.2 Microbiome as a risk factor for bronchial asthma

Zhou and colleagues identified a link between the diversity of gut microbiota and the control of allergic asthma via modulation of immune responses. Specifically, the presence of the bacterial strains Ruminiclostridium 6 and Candidatus Arthromitus has been associated with balancing the Th1/Th2 and Treg/Th17 cell ratios in eosinophilic asthma cases (74). Zhou et al. (2022) found 15 significant metabolism pathways, from gut microbiota, for eosinophilic asthma. These included malate, I-dihydroorotate, 1,5-anhydro-d-sorbitol and imidazoleacetic acid which were linked with Th17/Treg. Malate plays a vital role in the tricarboxylic acid (TCA) cycle, specifically in the synthesis of aspartate which is necessary for the proliferation of Th cells. There have been studies indicating that it can potentially interact with aspartate to control the activation of genes linked to T cell activation (75). Although there is currently no research specifically focused on malate’s role in allergic asthma disease, it is possible that it plays a role in the differentiation of Th cells, which is a crucial immune mechanism in allergic asthma disease. These research findings demonstrate that malate, l-dihydroorotate, imidazoleacetic acid, and 1,5-anhydro-d-sorbitol can serve as valuable biomarkers for diagnosing eosinophilic asthma (74). This discovery provides new insights into the disease and contributes to our understanding of its underlying mechanisms.

### 3.3 Barrier dysfunction in bronchial asthma

An array of stimuli can interfere with the integrity of the airway epithelial barrier. Allergens with protease activity, notably the principal allergen from house dust mites, Der p1, can directly compromise tight junctions (TJs) and indirectly via stimulation of protease-activated receptor-2 (76). Viruses like rhinoviruses and environmental pollutants such as diesel exhaust also disrupt TJs, enhancing the permeability of the airway epithelium and thus paving the way for inflammatory responses. Concurrently, various cytokines have been identified that can adversely affect the function of TJs (77, 78). A systematic review and meta-analysis has found a link between contact with cleaning agents and a higher incidence of asthma as it disrupts tight junctions by targeting proteins like occludin and ZO-1, augments paracellular permeability, and drives a TH2 immune response through the upregulation of IL-33 and TSLP (79).

### 3.4 Role of eosinophils in eosinophilic asthma pathogenesis

In recent years, the role of eosinophils in eosinophilic asthma has been increasingly recognized, extending beyond their traditional association with helminth immunity and allergies (80). Eosinophils have been found to contribute to type 2 inflammation, expanding their significance in the disease (81). Furthermore, eosinophil levels are significant indicators of disease severity and progression in eosinophilic asthma (82).

During an asthma attack, activated eosinophils release various proteins from their granules, contributing to the underlying mechanisms of the condition. These proteins, including major basic proteins, eosinophil peroxidase, eosinophil cationic protein, and eosinophil-derived neurotoxin, have toxic effects on the airway’s epithelial cells. Additionally, eosinophils secrete numerous inflammatory mediators such as cytokines (including interleukins IL-13 and IL-5), platelet-activating factors, growth factors (TGF-α and TGF-β), leukotrienes, thromboxane, and prostaglandins (83, 84). The release of these mediators contributes to the escalation of the inflammatory response, injury to the airway’s epithelial cells, airway hyperresponsiveness, excessive mucus production, and structural changes in the airways known as airway remodelling. This cascade of events also leads to bronchospasm, resulting in narrowed airways (83).

### 3.5 Th2-type immune responses in eosinophilic asthma

Th2-type immune responses, that involve the activation of type 2 helper T (Th2) cells and the secretion of specific cytokines, serve as a key factor in the pathogenesis of eosinophilic asthma (85, 86). After capturing allergens, dendritic cells situated within the epithelium proceed to process these allergens for antigen presentation to Th0 cells (87). Subsequently, The presence of IL-4 facilitates the differentiation of Th0 cells into Th2 cells as they become activated (88). Mast cells, basophils, and responding T cells themselves can produce IL-4 in response to low antigen concentrations (89). IL-4 is an important key factor in immunoglobulin class switching, and eosinophil trafficking (88).

Alongside IL-4, Th2 cells produce other key cytokines, including IL-5 and IL-13, which exert significant effects on the immune response (66, 68, 75). IL-5 plays a role in the development and release of eosinophils from bone marrow (69). Meanwhile, IL-13 contributes to the expansion of IgE-producing B cells and endothelial cells (70). Additionally, IL-13 collaborates with IL-4 to stimulate IgE production, induce nitric oxide generation, trigger goblet cell transformation and fibroblast proliferation, and prompt contractile responses and hyperplasia of airway smooth muscle cells (90). Therefore, the crucial role in promoting inflammation rich in eosinophils, which contributes to the distinctive characteristics of eosinophilic asthma, is played by both Th2 cells and the cytokines they release (86, 89).

### 3.6 Immunoglobulin E and eosinophilic asthma

Immunoglobulin E (IgE) has been widely acknowledged as a central player in the development of eosinophilic asthma, serving as the primary orchestrator of type 2 inflammation (91, 92). While IgE antibodies primarily contribute to the “early phase” of allergic reactions, their involvement in the “late phase” is considered less significant (91, 92).

The complex biological functions of IgE involve its interaction with specific receptors, including high-affinity (FcϵRI) and low-affinity CD23 (or FcϵRII) receptors, which influence the activities of various immune and structural cells implicated in chronic allergic inflammation (91, 92). Upon subsequent allergen exposure, the binding of IgE antibodies to high-affinity IgE receptors (FcϵRI) on mast cells, basophils, and dendritic cells prepares these cells for activation, resulting in the release of preformed proinflammatory autacoids (16, 70, 93). IgE also directly influences eosinophil functions, including their activation, eosinophil peroxidase release, heightened integrin expression, and the secretion of TNF-α (91, 92).

In the context of eosinophilic asthma, there is an increased IgE reactivity that specifically highlights its contribution to the progression of this type of asthma (94, 95). Significantly, approximately 80% of individuals with severe eosinophilic asthma exhibit elevated levels of specific IgE antibodies directed against perennial aeroallergens exceeding 0.35 IU/mL (71, 95).

### 3.7 Chemokines and eosinophil recruitment

The asthmatic lung produces a potent eosinophil chemoattractant called eotaxin, which is synthesized by various cell types and stimulated by IL-4 and IL-13, produced by Th2 lymphocytes (96, 97). In literature, Clara cells in the airway epithelium are reported as the primary source of eotaxin in the lungs (97). Specifically, IL-5 plays a distinct role in promoting eosinophil development in the bone marrow, assisted by IL-3 (83) and granulocyte-macrophage colony-stimulating factor (84), whereas the process of tissue recruitment hinges on the expression of eotaxins within the tissues (85). Eotaxin-1 and -2 are the primary chemokines responsible for attracting eosinophils to allergic airways (98). Th2 cells express chemokine receptors including CCR4, CCR8, CXCR4, and CCR3. Among these receptors, CCR8 plays a predominant role in inducing eosinophilia and airway hyper-responsiveness and may be elevated in the lungs and airways of individuals with eosinophilic asthma (85).

Apart from type 2 cytokines, eosinophils also possess the capability to generate and release type 1 inflammatory cytokines and chemokines. Eosinophils can secrete chemokines like CCL5/RANTES, CCL11/eotaxin, and CCL3, which attract Th2 lymphocytes to the inflammation site, thus contributing to the perpetuation of the inflammatory cycle (10).

### 3.8 Emerging role of innate lymphoid cells in eosinophilic asthma

ILCs, a recently discovered type of immune cells, possess lymphoid morphology but lack antigen receptors, as discussed in the section of CRSwNP (99). Among the three subtypes of ILCs, ILC2s are non-B/non-T cells that release IL-5 and IL-13 (100).

The airway epithelium releases epithelial-derived cytokines, including IL-33, IL-25, and thymic stromal lymphopoietin (TSLP), which activate downstream ILC2s. Consequently, ILC2s become a primary source of IL-13 and IL-5. Notably, IL-5 plays a crucial role in eosinophil production, promoting the generation of mature eosinophils in both the bone marrow and airways. The subsequent migration of eosinophilic progenitors and mature eosinophils to the lungs contributes significantly to the pathogenesis of eosinophilic asthma (101).

The degree of the disease’s severity is associated with the abundance of type 2 innate lymphoid cells (ILC2s); patients with severe asthma exhibit higher counts of IL-5 and IL-13 secreting ILC2s in their sputum compared to those with milder forms of the condition (102). A significant discovery in this study is the distinct contrast in the inherent type 2 responses observed among individuals with allergic asthma and those with allergic rhinitis. Hence, the augmentation of innate type 2 immunity and the proliferation of ILC2s are improbable indicators for Th2-type immune responses in a broad sense. It is speculated that ILC2s and their resulting cytokines, namely IL-5, IL-13, and tissue growth factors, manifest a more significant impact on allergic asthma compared to allergic rhinitis (102).

Research indicates a significant interaction between ILC2s and the airway epithelium in asthma’s development. IL-13-producing ILC2s, which are triggered by alarms released from the epithelium, have been shown to compromise the integrity of bronchial epithelial cells, both in laboratory cultures and animal models. This process perpetuates type 2 inflammatory reactions within the airways, contributing to the occurrence of asthma exacerbations. Based on the similarities in transcription factor expression, cytokine secretion, and localization, it appears that ILC2s can contribute to epithelial barrier breakdown through IL-13 production. Furthermore, ILC-2 can stimulate dendritic cells (DCs) to produce CCL17 via IL-13, which facilitates Th2 cell chemotaxis to local mucosa. However, recent studies suggest that once T cells are activated, ILC2s may not be necessary (103).

Genetic association studies have uncovered those variations within the IL33 gene, the ST2 (IL1RL1) receptor, the ROR-α (RORA) transcription factor, and IL13—all pivotal in the development and activation of ILC2s—are linked to asthma with a high presence of type 2 helper T cells. A recent epigenomic investigation has identified robust associations between gene regulation in ILC2s and the hereditary components of asthma, underscoring the influential role of ILC2s in individuals with allergic asthma (104). Based on the similarities in transcription factor expression, cytokine secretion, and localization between ILC2s and Th2 cells, Sugita’s mouse data suggest that ILC2s may contribute to the disruption of the epithelial barrier through the production of IL-13. Nonetheless, the dispensability of ILC2s arises once T cells have undergone activation. These findings, combined with the research conducted by Sugita et al., propose that the partnership between ILC2s and Th2 cells plays a role in driving inflammatory conditions like asthma and CRS w NP. Furthermore, it has been documented that ILC2s play a role in the recruitment of memory Th2 cells. Interestingly, studies have shown that mice without ILC2s but with Th2 cells exhibit decreased levels of Th2 cytokines and IgE, underscoring the significance of ILC2s in facilitating the transition from innate to adaptive Th2 immune responses. However, there are still unanswered questions surrounding this topic that need to be further explored. To address questions regarding the correlation between disease severity or exacerbation and the number or activity of circulating ILC2s, it is evident that further clinical studies with larger sample sizes and different study designs are required.

### 3.9 Cellular interactions in eosinophilic asthma

In the case of eosinophilic asthma, eosinophils, Th2 lymphocytes, mast cells, and epithelial cells are key players involved in a complex network of interactions (70, 85). Eosinophils are recruited to the airways by chemokines like eotaxins and cytokines such as IL-5. Once activated, eosinophils release inflammatory mediators that exacerbate airway inflammation and hyper-responsiveness (83). Th2 lymphocytes play a role in the recruitment and activation of eosinophils by producing cytokines like IL-4, IL-5, and IL-13 (89). Mast cells, when exposed to allergens, release histamine and other pro-inflammatory molecules, triggering bronchoconstriction and attracting eosinophils (105). Epithelial cells in the airways can also contribute to eosinophil recruitment by producing eotaxins, cytokines, and other chemokines (85).

**Figure 1:**
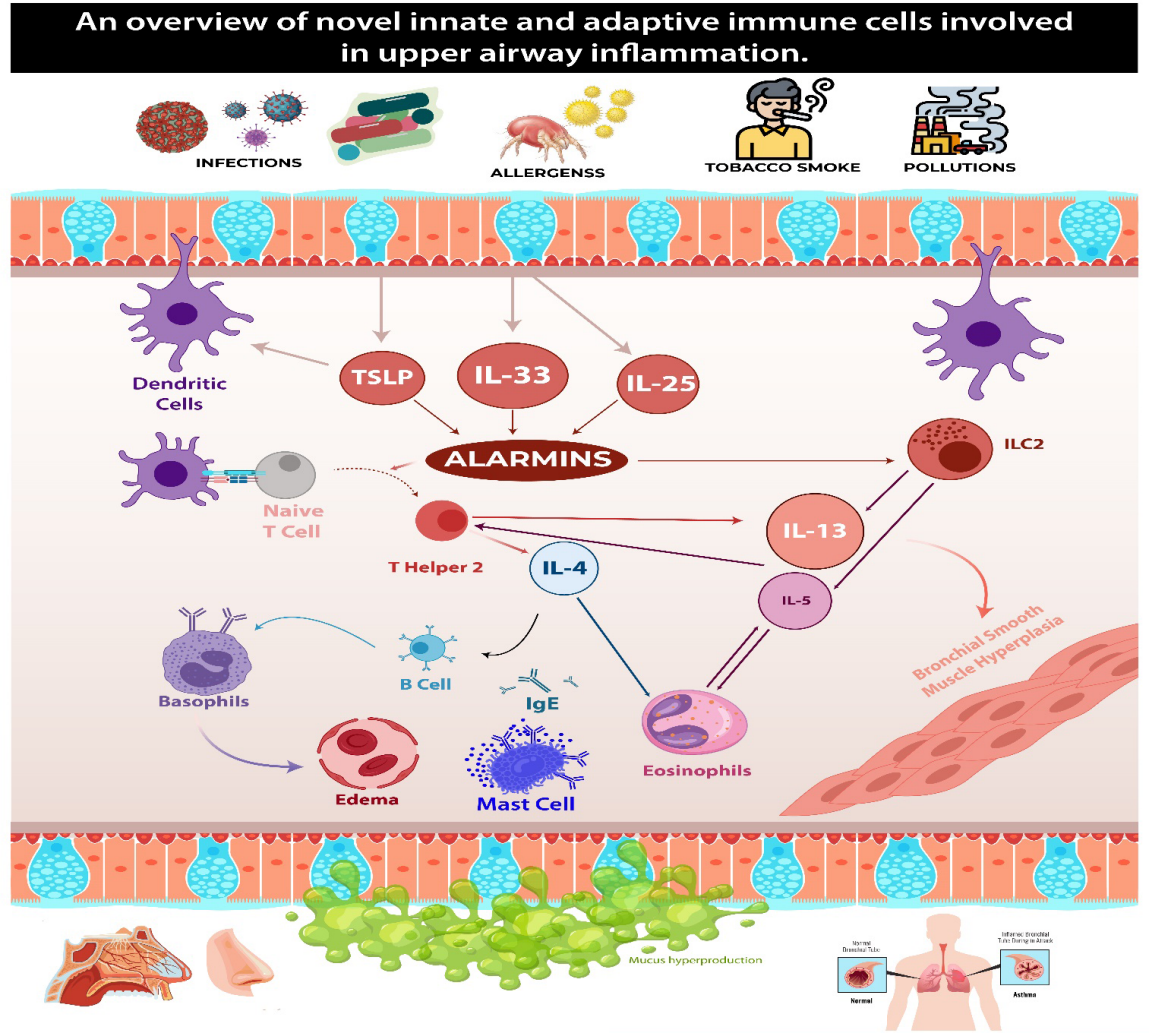
This figure demonstrates the common pathways in type two inflammation seen in patients with CRSwNP and eBA. Upon encountering environmental triggers, epithelial cells release interleukin-33 (IL-33), thymic stromal lymphopoietin (TSLP), and IL-25. These substances activate group 2 innate lymphoid cells (ILC-2), which in turn begin and sustain eosinophilic inflammation by secreting type 2 cytokines. ILC-2 also prompt dendritic cells (DCs) to secrete CCL17 through IL-13, thereby attracting Th2 cells to the local mucosa. Dendritic cells play a crucial role in steering naïve T cells toward a T helper 2 (Th2) response, facilitating their expansion. Concurrently, ILC-2s induce T cell responses and enhance B cell antibody production. Th2 cells generate IL-4, which triggers a class switch and the production of IgE in B cells. The cross-linking of IgE results in the degranulation of mast cells. Additionally, Th2 cells contribute to the mobilization and activation of eosinophils by producing the cytokine IL-5, which contributes to tissue inflammation.

In conclusion, the immunopathology of eosinophilic asthma involves interactions between genetic factors, environmental triggers, and the immune system, particularly of type 2 inflammation. **Figure 1** summarizes type two inflammation cascade in CRSwNP and eBA.

### 3.10 The unified inflammatory airway diseases; similar risk factors, common type 2 inflammation process and a shared epithelial barrier

Studies suggest that different inflammatory airway conditions might be interlinked, illustrating the concept of ‘unified airway diseases’. A study by Marcus and colleagues involving 356 individuals identified that asthma was most frequently found in patients with Aspirin-Exacerbated Respiratory Disease (AERD), affecting all individuals in the group (100%), and Chronic Rhinosinusitis with Nasal Polyps Not Otherwise Specified (CRSwNP NOS), impacting 37.1%. Conversely, Allergic Fungal Rhinosinusitis (AFRS) and Central Compartment Atopic Disease (CCAD) displayed much lower incidences, at 19.0% and 17.1% respectively (25). Typically, asthma is perpetuated by allergic sensitization, and allergic asthma constitutes the most prevalent diagnosed form of asthma, encompassing over 60% of cases (25).

The primary cellular component of the airways is the ciliated epithelial cell, which develops from club cells or airway basal cells. The differentiation of basal cells into ciliated epithelial cells is intricately controlled by the Notch signaling pathway. Elevated levels of Notch drive the transition towards mucus-producing goblet cells. Type 2 cytokines, such as IL-4 and IL-13, are known to activate the Notch pathway, which correlates with an augmented presence of goblet cells in conditions like asthma and chronic rhinosinusitis (CRS) (106). These ciliated cells, adorned with abundant cilia, play a crucial role in mucociliary clearance, and disruptions in their function are observed in conditions like asthma and allergic rhinitis (AR).

Goblet cells, which are specialized for secretion, are filled with vesicles containing mucins and surfactant proteins. Their primary role is to release mucins such as Muc5AC and Muc5B onto the airway surfaces to capture external particles. The equilibrium between mucin secretion and its removal is delicate; disturbances in this balance, often caused by cytokines like IL-4 and IL-13, are associated with diseases such as asthma, AR, and CRS. Distinct subtypes of goblet cells have been identified, each with unique functional properties (107).

Recent research has uncovered unique subsets of basal cells that exhibit specific gene expressions, including IL-33, thymic stromal lymphopoietin (TSLP), and an IL-4/IL-13 gene signature. These subsets have been found in bronchial biopsies from asthma patients and nasal polyps from individuals with CRS with nasal polyps (CRSwNP), suggesting a deeper complexity in the cellular mechanisms of these diseases (108).

A variety of environmental insults trigger both the innate and adaptive branches of the immune system to shift towards a type 2 immune response. The research by Celebi Sözener et al. recognizes various environmental elements as contributing to the impairment of the epithelial barrier. Particulate matter (PM2.5 and PM10) and diesel exhaust particles are implicated in the disruption of key proteins that maintain tight junction integrity, such as occludin, claudin-1, and ZO-1, leading to decreased expression of claudin-1 in airway epithelial cells. These particles also cause a separation of occludin from ZO-1 and provoke an upsurge in reactive oxygen species (ROS), affecting the structural components of cells such as cytokeratin, filaggrin, and E-cadherin (109).

Microplastics, due to their distinct three-dimensional structures, can infiltrate cell membranes and influence lipid distribution within the lipid bilayer. They also have a role in initiating cell apoptosis, altering the metabolic landscape of airway epithelial cells, and promoting oxidative stress (109). Moreover, enzymatic allergens are recognized for initiating non-IgE mediated responses via activation of proteinase-activated receptors, leading to the breakdown of critical barrier proteins and increased epithelial permeability.

The airway epithelium is not only instrumental in initiating this response but also becomes a secondary site of inflammation. The repeated activation of eosinophils, along with basophils and mast cells, leads to edema and significant structural changes in tissues. Consequently, inflammation and damage to the epithelium are central to the development of symptoms observed in skin and respiratory diseases. It has been shown that IL-13 disrupts bronchial epithelial integrity by affecting tight junctions in individuals with asthma (103) (103). The airway epithelium actively influences immune responses by secreting alarmins such as TSLP, which, when released from nasal polyps, provokes a heightened IL-5 response from mast cells. This effect is mirrored in the lungs of asthmatic patients who exhibit increased levels of TSLP compared to healthy individuals (110). After exposure to allergens, IL-25 is markedly upregulated in the bronchial mucosa and nasal polyps of CRSwNP patients (57, 111). Elevated levels of IL-25 in nasal polyps are also indicative of a positive response to corticosteroid treatment (112). In asthma, higher bronchial IL-25 expression correlates with increased eosinophil activity, both locally in sputum and systemically in the bloodstream (113). IL-33, belonging to the IL-1 cytokine family, also plays a role in type 2 inflammatory responses and is secreted by both epithelial cells and bronchial smooth muscle cells. Its release significantly triggers type 2 innate immune mechanisms in the airways, leading to the recruitment of eosinophils. Genetic research has drawn connections between IL-33 levels and the occurrence of nasal polyps in CRSwNP (114).

The initiation of the immune response is influenced not just by epithelial signals but also by the actions of professional antigen-presenting cells like dendritic cells (DCs). In the context of asthma, airway DCs are vital in guiding naïve T cells to commit to a T helper 2 (Th2) response and multiply thereafter. While CD4+ T cells that secrete IL-4, IL-5, and IL-13 are well-known for their production of key type 2 cytokines, Group 2 innate lymphoid cells (ILC2s) also play a significant role in generating these cytokines. ILC2s, especially when activated by TSLP, have shown resistance to corticosteroids, which enables them to continue producing type 2 cytokines despite therapeutic intervention (115). This resistance is mirrored in eosinophilic nasal polyposis, where a higher presence of ILC2s in the mucosa correlates with a response to corticosteroids (116, 117). B cells, in the presence of IL-4, transition to producing IgE, essential for priming basophils and mast cells to react to allergens. This IgE, upon binding to high-affinity Fc receptors on these cells, primes them for activation, which occurs when allergens later trigger the IgE, leading to the release of stored cytokines. Histamine, alongside other substances like tryptase, prostaglandins, and leukotrienes released during this activation, disrupts vascular integrity and leads to swelling. These substances also aid in attracting more inflammatory cells to the area. Environmental factors can stimulate increased IgE production. Moreover, IgE serves a crucial function in recognizing low levels of allergens and stimulating the adaptive immune system toward a type 2 response. Antigen-presenting cells, including DCs, B cells, and basophils, take in IgE-allergen complexes via both high (FcϵRI) and low (CD23) affinity IgE receptors and present the allergens to T cells, further driving the immune response (118).

Elevated levels of eosinophils in the bloodstream are closely linked with increased rates of flare-ups in a subset of patients with severe asthma who are prone to exacerbations (119). Eosinophil basic protein secretion is a distinctive aspect of type 2 inflammatory diseases, contributing to conditions such as increased airway sensitivity and the development of nasal polyps (120). Addressing the common underlying processes of these inflammatory airway conditions presents a promising strategy for concurrently managing multiple diseases, offering a compelling treatment target for affected patients.

## 4 Immunopathology of other eosinophilic airway diseases

Eosinophilic asthma derives its name from the presence of eosinophilic infiltration alongside asthma symptoms (121). This distinctive mechanism of eosinophilic inflammation is also observed in several other pathologies (80, 122–124). However, for our discussion, we have chosen to focus on eosinophilic granulomatosis with polyangiitis (EGPA) and allergic bronchopulmonary aspergillosis (ABPA) only.

EGPA is a systemic condition characterized by eosinophilic necrotizing vasculitis, whereas ABPA is an allergic disorder associated with intensified reactivity to Aspergillus (125, 126). ABPA primarily affects the respiratory tract, whereas EGPA is a systemic eosinophilic disease. However, these conditions exhibit shared diagnostic criteria such as asthma, peripheral blood eosinophilia, and elevated serum IgE levels, indicating a potential involvement of common immune mechanisms in their development (125, 126).

### 4.1 Immunopathology of eosinophilic granulomatosis with polyangiitis

EGPA, previously referred to as Churg-Strauss syndrome, is an intricate inflammatory condition characterized by eosinophil-rich granulomatosis. It commonly affects the respiratory tract and also involves necrotizing eosinophilic vasculitis, primarily impacting small to medium-sized blood vessels. The disorder is linked to significant eosinophilia in the blood or tissues, and around 30%–40% of patients exhibit the presence of antineutrophil cytoplasmic antibodies (ANCA) (71, 95, 96). EGPA poses a life-threatening risk and is considered a rare form of systemic vasculitis, estimated to affect approximately globally 1.22 cases per million person-years (124).

EGPA commonly sequentially progresses through three distinct phases. The allergic phase is marked by the presence of asthma, allergic rhinitis, and sinusitis. The eosinophilic phase is characterized by the predominant pathological feature of eosinophilic infiltration in various organs, such as the lungs, heart, and gastrointestinal system. Lastly, the vasculitic phase manifests with symptoms including purpura, peripheral neuropathy, and end symptoms (125).

**Figure 2:**
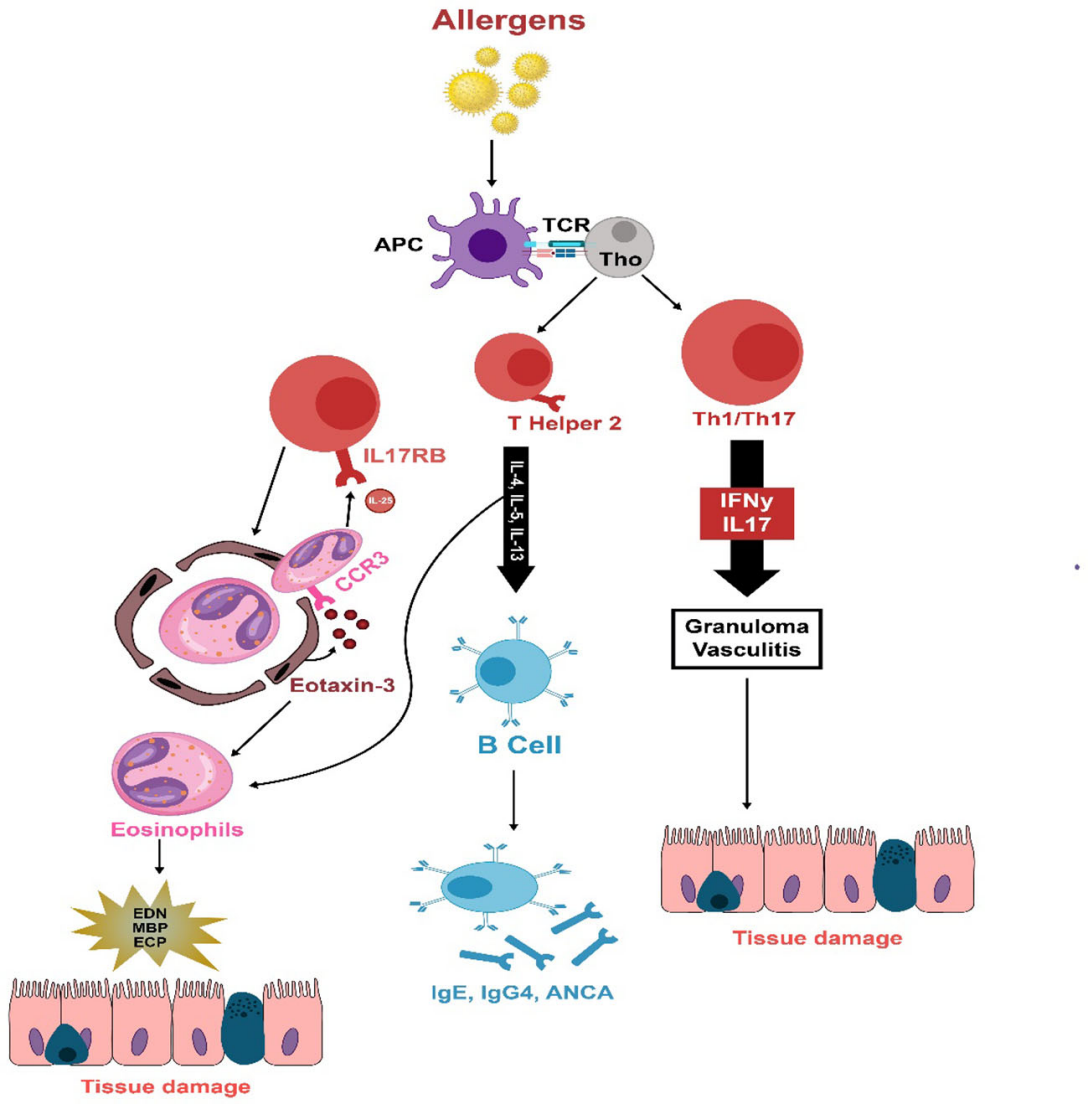
The immunopathology of EGPA is thought to be initiated by unidentified allergens that provoke an adaptive immune reaction in susceptible individuals. T cells secrete cytokines associated with Th1 (IFN-γ), Th17 (IL-17), and Th2 (IL-4, IL-13, IL-5) responses, which activate eosinophils. A strong Th2 response stimulates B cells to produce IgG4, IgE, and ANCA. Eotaxin-3, which is increased and released, draws eosinophils to the endothelium and deeper into tissues. These eosinophils sustain T-cell activation by emitting IL-25. Eosinophils also release damaging agents such as eosinophil-derived neurotoxin, major basic protein, and eosinophil cationic protein when they degranulate, leading to tissue and vessel damage, necrosis, and fibrosis.

The exact pathogenesis of EGPA remains incompletely comprehended; however, it is likely attributed to the complicated interplay between T cells, B cells, and eosinophils (127, 128). The underlying mechanism involves the activation of adaptive immune response by yet unidentified allergens. T cells release cytokines associated with Th1 (IFN-γ), Th17 (IL-17), and Th2 (IL-4, IL-13, IL-5) responses, stimulating eosinophil activation (123, 129). The robust Th2 immune response triggers B-cell activation, leading to the production of IgG4, IgE, and ANCA. Elevated expression and release of Eotaxin-3 play a role in directing eosinophils toward the endothelium and surrounding tissues. Consequently, activated eosinophils perpetuate a cycle of T-cell activation through the secretion of IL-25. The localized degranulation of these activated eosinophils eventually leads to tissue and vessel damage, necrosis, and fibrosis (123). **Figure 2** summarizes the key inflammatory process in EGPA.

#### 4.1.1 Role of T cells

EGPA is commonly characterized as a Th2-driven hyper-inflammatory response, with IL-4, IL-5, and IL-13 being the main cytokines associated with a Th2 immune response. Patients with EGPA often exhibit elevated production of IL-4 and IL-13 by peripheral T-cell lines (123, 129). Furthermore, elevated concentrations of IL-5 are detectable in the serum. and bronchoalveolar lavage (BAL) fluid of individuals with active EGPA (130).

In addition to the Th2 subset, emerging research suggests that other T-cell subsets, particularly Th17 cells might contribute to the development of EGPA (131). During active disease, there is an increased frequency of Th17 cells that secrete higher levels of IL-17A, underscoring their potential contribution to the disease process (130). Furthermore, disruptions in the balance and function of regulatory T cells, which are responsible for suppressing Th17 cells, have been linked to EGPA, further supporting the role of Th17 cells in the pathogenesis of the disease (132, 133).

#### 4.1.2 Role of B cells

It is becoming more widely acknowledged that B lineage cells in EGPA patients display a dysregulation in various receptors and ligands, such as B cell activating factor (BAFF) and a proliferation-inducing ligand (APRIL), as well as downstream effector molecules, including Bruton’s tyrosine kinase (BTK) and spleen tyrosine kinase (SYK) (132, 133). These changes could potentially play a role in the development of the disease by promoting heightened differentiation towards plasmablasts and plasma cells, increased production of IgG4, auto-antibodies and cytokines, and the facilitation of antigen presentation to T cells, thereby contributing to disease pathogenesis (132, 133).

#### 4.1.3 Role of eosinophils

Eosinophils play a pivotal role in the inflammation associated with EGPA. They are the predominant cell type involved in both circulating and tissue eosinophilia, which are characteristic features of the disease, and their dysregulated activation is linked to an aberrant Th2 response (131). In EGPA, eosinophils are prominently present in the inflamed tissues, driven by the skewed Th2 environment (130). The process of eosinophil maturation, activation, and longevity is shaped by the crucial cytokine IL-5, while the presence of eotaxin-3, which is generated by both endothelial and epithelial cells, is presumed to play a role in attracting eosinophils to the afflicted tissue (134, 135).

Eosinophils exert their pathogenic influence by releasing granule proteins, such as eosinophil major basic protein (MBP), eosinophil-derived neurotoxin (EDN), and eosinophil cationic protein (ECP). These cytotoxic enzymes directly destroy tissues (96). Furthermore, activated eosinophils discharge IL-25, which amplifies Th2 cytokine production and may potentially sustain the ongoing hyperinflammatory process (136).

#### 4.1.4 Other contributors

Apart from the participation of T cells, B cells, and eosinophils, several additional elements play a role in EGPA’s development. It’s worth noting that antineutrophil cytoplasmic antibodies (ANCA) have been identified in 40% of patients. Furthermore, elevated levels of IgG4 have been noted in EGPA and are linked to the extent of organ involvement and disease activity (130).

Chemokines, specifically eotaxin-3 (CCL26), play a significant role in EGPA pathogenesis by attracting eosinophils to sites of inflammation. Many investigations have documented heightened concentrations of eotaxin-3 in the serum of individuals with active EGPA, and this is closely associated with eosinophil counts, overall IgE levels, and markers of acute-phase reactions. Moreover, immunohistochemical assessments have unveiled notable eotaxin-3 expression in endothelial and inflammatory cells within affected tissues of individuals with active EGPA (134, 135). Additionally, another Th2 chemokine, ILC2, has been identified in both tissue infiltrates and sera of EGPA patients (132). Furthermore, recent studies have indicated that *in vitro* stimulation of T cells from EGPA patients results in the production of significant amounts of interferon-gamma (INF-γ), which enhances Th1 immune responses (129).

### 4.2 Allergic bronchopulmonary aspergillosis

ABPA is a condition marked by a hypersensitivity reaction in the lungs caused by the fungal infection of Aspergillus fumigatus. It involves an initial allergic response and subsequent lung damage, which occurs with repeated exposure to Aspergillus antigens. The ongoing colonization of the airways by the fungus is responsible for the progression of ABPA (137). Academic investigations imply that approximately 2.5% of adults with asthma experience ABPA, falling within a spectrum of 0.7-3.5% (138). Diagnostic criteria have been proposed for ABPA, including a history of asthma, specific IgE levels for A. fumigatus higher than 0.35 kUA L−1, total IgE levels greater than 1000 IU/mL, and at least two of the following criteria: temporary or persistent pulmonary infiltrates, peripheral blood eosinophil count exceeding 500 cells/μL, and the presence of serum precipitins or IgG antibodies against A. fumigatus (139).

**Figure 3:**
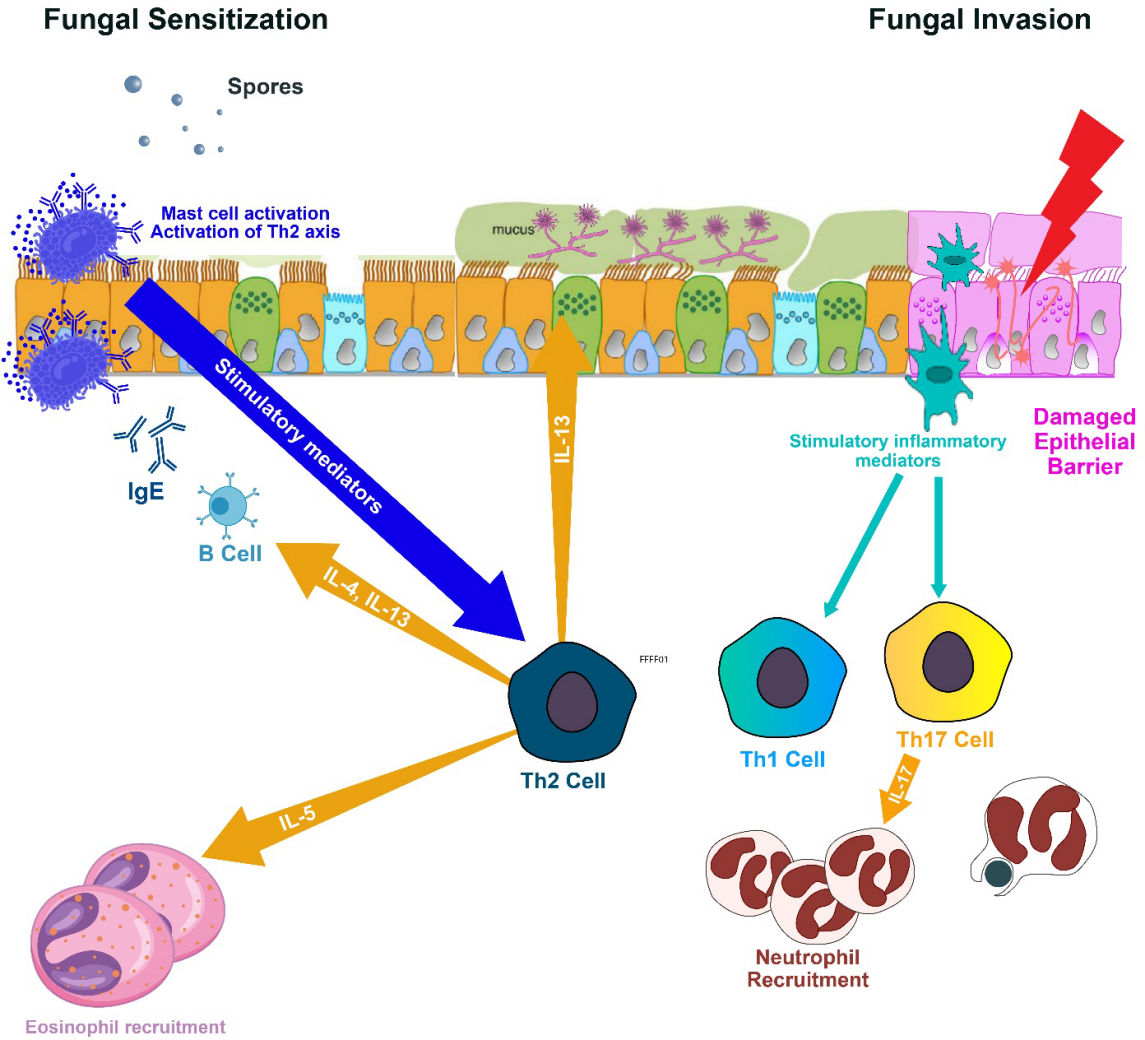
Spores of Aspergillus fumigatus, once inhaled, adhere to bronchial cells and develop into fungal networks. These fungi then secrete proteases and allergenic compounds that incite inflammation, excessive mucus, and harm the airway surfaces, compromising mucociliary function and aiding in additional fungal proliferation. Inflammatory signals from epithelial cells mobilize eosinophils and neutrophils to the airways, causing obstruction. Antigen Presenting Cells polarizes naïve Th0 to Th1, Th17 and Th2. The Th1 immune response is pivotal in eradicating the infection, but the Th2 response perpetuates fungal residency, associated with allergic aspergillosis. Th2 cells elicit the release of IL-4 and IL-13, leading to IgE antibody production by B cells and IL-5, which prompts eosinophils to inflict airway damage. Additionally, the Th17 response recruits granulocytes but can also contribute to damage due to immune responses. IL, interleukin; and Th, T lymphocyte helper.

To comprehend the immunopathology of ABPA, we have categorized it into subsequent subsections based on the order of appearance (140). **Figure 3** Summarizes the key inflammatory process in ABPA.

#### 4.2.1 Antigen presentation and activation of Th2 immune responses

While exposure to many A. fumigatus conidia can potentially lead to ABPA, not all individuals with asthma develop the condition (139, 140). In susceptible individuals, The onset of pathogenesis starts when A. fumigatus spores are inhaled and subsequently sprout into hyphae deep inside the bronchi (140). Antigen-presenting cells (APCs) carrying human leukocyte antigen (HLA) DR5 or HLA DR2 process these allergens and present them to helper T cells within the bronchoalveolar lymphoid tissue (BALT) (141). Research has underscored the role of different T-helper subgroups in ABPA. Th1, Th2, and Th17, the three major T-helper lineages, have all been identified as having important contributions. The Th1 response is essential for effectively clearing fungal infection, while the Th2 responses aid in the sustained presence of the fungus and are linked to allergic types of aspergilloses. The Th17 response serves a dual role, playing a crucial role in the recruitment of granulocytes while also potentially contributing to detrimental immunopathology (142).

Amongst these various subsets of T-helper cells, there is a prevailing Th2 response that is particularly involved and is believed to be impacted by genetic factors (143). Furthermore, the activation of T cells results in the release of inflammatory cytokines, including IL-4, IL-5, and IL-13, which further promote the Th2 response (144).

#### 4.2.2 Elevated levels of serum IgE

Individuals with ABPA show increased sensitivity to IL-4, resulting in the spontaneous production of higher amounts of IgE, IgG, and IgA antibodies against A. fumigatus antigens by B cells (113). When IgE binds to mast cells and causes the cross-linking of IgE molecules, it triggers mast cell degranulation, which subsequently results in the discharge of histamine, leukotrienes-B (LTB), and various other mediators (141).

#### 4.2.3 Eosinophilia in allergic bronchopulmonary aspergillosis

In ABPA, eosinophils have a significant function as effector cells and are recruited by various cytokines, including LTB4, platelet-activating factor, eotaxin, monocyte chemoattractant protein 3 (MCP-3) (145). Eosinophils have Fc receptors designed to bind with IgE, IgG, and IgA antibodies. Specific IgG, IgA, and IgE antibodies targeting A. fumigatus attach to these receptors and undergo cross-linking, triggering eosinophil degranulation. This results in the release of eosinophil granules causing damage in airway epithelial cells and exacerbation of bronchial inflammation (146).

Apart from blood eosinophilia, a characteristic feature of ABPA is the massive accumulation and clustering of eosinophils in the bronchial lumen, leading to bronchial impaction. This phenomenon has been described using various terms depending on the context, such as “allergic mucin,” “high attenuation mucus,” or “allergic mucus plugs” (147, 148).

## Conclusion

In summary, the underlying mechanisms involved in CRSwNP, eosinophilic asthma, EGPA, and ABPA share common immunopathological features. In CRSwNP, the dysfunction of the epithelial barrier is driven by disrupted inflammatory pathways, alterations in airway lining cells, and communication with mast cells, resulting in persistent type 2 inflammation. Eosinophilic asthma arises from interactions among genetic factors, environmental triggers, and the immune system, leading to type 2 inflammation mediated by Th2 cells, B cells, mast cells, and ILCs2. EGPA and ABPA involve the activation of adaptive immune responses, with T cells releasing cytokines associated with Th1, Th2, and Th17 responses, leading to eosinophil activation, tissue damage, and associated complications. **Table 2** outlines a comparison of key inflammatory markers in eosinophilic airway diseases. Understanding these shared immune mechanisms is essential for the formulation of precise therapeutic approaches aimed at managing these conditions effectively.

**Table 2:** Comparison of immunological markers eosinophilic airway diseases.

Feature/Disease	CRSwNP	Eosinophilic Asthma	EGPA	ABPA
Definition	Inflammatory ailment in nasal mucosa and sinuses	Asthma subtype with high eosinophil levels	Multisystem inflammatory disease affecting respiratory tract and small to medium blood vessels	Hypersensitive response to Aspergillus fungus
Prevalence	5-15% in general population	Approx. half of asthma cases	1.22 cases per million person-years	0.7-3.5% in adults with asthma, 6-8- 12% in cystic fibrosis
Predominant Type of Inflammation	Type 2 inflammation	Type 2 inflammation	Eosinophil-rich granulomatosis	Allergic reaction
Key Cells Involved	Eosinophils, T cells Th2 (Th17/ Treg imbalance)	Eosinophils, Th2 cells, ILC2s	Eosinophils, T cells (Th1, Th2, Th17)	Eosinophils, Th2 cells
Epithelial Barrier Dysfunction	Yes	–	–	–
Role of Eosinophils	Central in inflammation	Indicator of disease severity	Predominant in inflammation	Effector cells in lung damage
Cytokines and Chemokines	IL-4, IL-5, IL-13, IL- 17, IFN-γ	IL-4, IL-5, IL-13, eotaxins	IL-4, IL-5, IL-13, eotaxin-3	IL-4, IL-5, IL-13
Predominant Immune Response	Type 2, Th2 predominance	Type 2, Th2 predominance	Th2-driven, Th1 and Th17 involvement	Th2 response predominance
IgE Levels	–	Elevated in severe cases	Elevated, associated with ANCA	Significantly elevated
Associated Conditions	Asthma, especially type 2	–	Asthma, sinusitis, allergic rhinitis, purpura, neuropathy	Asthma/ cystic fibrosis
Treatment Targets	Type 2 inflammation, epithelial function	Type 2 inflammation, eosinophil levels	Th2 cytokines, eosinophil activation	Th2 immune response, fungal colonization

CRSwNP, chronic rhinosinusitis with nasal polyps; eBA, eosinophilic bronchial asthma; EGPA, Eosinophilic Granulomatosis with Polyangiitis; and ABPA, allergic bronchopulmonary aspergillosis; IL, interleukin; IFN-γ, interferon-gamma; ILC-2, innate lymphocyte-2; and Th, T lymphocyte helper.

## Author contributions

MA: Writing – review & editing, Writing – original draft, Validation, Methodology, Data curation, Conceptualization. SA: Writing – review & editing, Writing – original draft.
